# PMO’s protective wrap: Unraveling its surfactant-mediated stabilization for next-generation antisense therapies

**DOI:** 10.1016/j.omtn.2026.102897

**Published:** 2026-03-25

**Authors:** Swrajit Nath Sharma, Abhishek Gupta, Surajit Sinha

**Affiliations:** 1 School of Applied and Interdisciplinary Sciences, Indian Association for the Cultivation of Science, Kolkata 700032, India

## Introduction

The rapid ascent of phosphorodiamidate morpholino oligonucleotides (PMOs) has fundamentally transformed the therapeutic landscape for rare genetic disorders such as Duchenne muscular dystrophy (DMD). At the vanguard of this revolution are the drug-like properties of PMOs, including high solubility, remarkable resistant to metabolic degradation, and a safer toxicity profile, which make them potent candidates for precision genetic medicine. Unlike traditional DNA and RNA therapeutics—defined by their negatively charged phosphate backbones—PMOs are uniquely charge-neutral. Consisting of a neutral phosphorodiamidate backbone and a six-membered morpholino ring, their structural features confer high aqueous solubility, metabolic stability, and low immunogenicity compared with other charged oligonucleotides. Clinically validated PMOs, such as eteplirsen and golodirsen (targeting DMD exon 51 and exon 53, respectively), have received U.S. Food and Drug Administration approval in recent years. These agents function via steric hindrance during pre-mRNA splicing, thereby restoring dystrophin protein expression in patients with DMD.^1^^,^^2^ Nonetheless, PMOs exhibit amphiphilic traits due to nucleobase hydrophobicity and backbone hydrophilicity, which can lead to interfacial adsorption and aggregation at high concentrations.^3^ To mitigate these risks, the pharmaceutical industry often employs non-ionic surfactants.^4^ Indeed, neutral surfactants such as N,N-bis-(3-D-gluconamidopropyl)-cholamide (BigCHAP) and N,N-bis-(3-D-gluconamidopropyl)-deoxycholamide (Deoxy-BigCHAP) have previously been reported to improve the delivery and exon-skipping efficiency of PMOs both *in vitro* and *in vivo,* underscoring the importance of surfactants.^5^ However, the precise nature of the structure-function relationship and the molecular dynamics (MD) interactions between these surfactants and PMOs remains largely uncharacterized. In a recent study, Kliuchnikov et al. provided a landmark analysis of how common pharmaceutical surfactants, specifically Polysorbate 20 and 80, interact with PMOs to prevent aggregation, regulate adsorption at interfaces, and stabilize these vital therapeutic agents.^6^

## Merits and key findings

**Figure 1:**
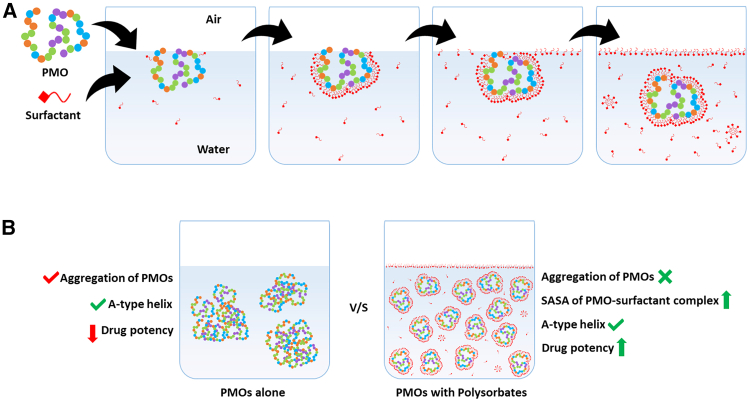
Surfactant-PMO interactions and their effects on physiochemical properties of PMOs Graphical representation of (A) different stages of interaction between surfactants and PMOs in the solution state with increasing surfactant concentration. (B) Comparison of PMO aggregation in the presence and absence of Polysorbates.

By combining surface tension measurements and circular dichroism (CD) spectroscopy with MD simulations, the researchers probed the interactions between therapeutic 25-mer and 30-mer PMOs (targeting DMD exons 53 and 51, respectively) and the non-ionic surfactants Polysorbate 20 and 80. They demonstrated that PMOs interact with surfactants in a remarkably “protein-like” manner. Using surface tension titration, they identified distinct, staged regions of interaction that mirror the behavior of complex proteins. As surfactant concentration escalated, the molecules moved from occupying the air-water interface to “loading” onto the PMO molecules, eventually displacing the PMOs from the surface entirely to form stable 1:1 complex. Interestingly, in the presence of PMOs, the critical micelle concentration (CMC) values of Polysorbate 20 and 80 were observed to increase compared with those of the Polysorbates alone, thereby indicating an interaction between the PMOs and the surfactants. Importantly, the MD simulations provided a high-resolution view of Polysorbate 20 and 80 acting as molecular chaperones. During transient instances of PMO unfolding toward extended conformations, the surfactants were observed to facilitate a refolding process back into a more compact, energetically favorable structure. While the interaction energy is distributed across the entire sequence, certain residues act as high-affinity hotspots that sustain persistent contacts with the surfactant tails. A striking finding of the research is the consistent preference of the surfactants’ long hydrophobic tails for purine bases. Due to their larger, two-ring planar systems, purines provide a more expansive hydrophobic surface area compared with single-ring pyrimidines. The long hydrophobic tails seek out these flat hydrophobic faces to establish energetically favorable interactions. The simulations revealed that the specific folding patterns of individual conformers dictate these “interactive” zones. In the 25-mer, position 10 (G) was identified as a critical site for Polysorbate 80, maintaining persistent contacts. In the 30-mer, a cluster of residues, including 16 (A), 17 (G), and 23 (A), exhibited the strongest interactions with Polysorbate 80. Because Polysorbate 20 has a shorter aliphatic chain (11 carbons) compared with Polysorbate 80 (17 carbons), its binding sites are slightly less selective for purines, often interacting with residues such as 11 (T) and 14 (T) in the 25-mer. Despite these strong localized bonds, the surfactants do not disrupt the molecular structure. The secondary structure, measured by base-pairing and base-stacking counts, remained nearly identical to that of the PMO alone in solution, as confirmed by CD spectroscopy. Even in the presence of a large surfactant excess, the PMO’s essential chirality and “A-type” helical structure remained unchanged. This indicates that surfactants provide a protective wrap that stabilizes the tertiary structure without interfering with the molecular machinery required for antisense activity. The significance of this work for the field of translational medicine cannot be overstated. By elucidating the energetics and interaction dynamics of PMO-surfactant complexes, the study shifts the field from empirical formulation to a precise, rational drug design framework (Figure 1).

## Impact and outlook

The present paper contributes to the rational design of RNA-mimic therapeutics by clarifying how surfactants (e.g., Polysorbates) stabilize PMOs without compromising their functional structure. It bridges a gap between molecular biophysics and pharmaceutical formulation science, offering insights that could improve the following:

**Stability:** The data explains how surfactants reduce the solvent-accessible surface area (SASA) of PMOs, making them less prone to intermolecular interactions and aggregation during formulation.

**Increased Solubility:** While individual PMO surfaces are shielded, the overall PMO-surfactant complex becomes more solvent-exposed, effectively increasing the drug’s solubility and potentially improving its shelf-life.

**Framework for New Chemistries:** The enhanced biophysical properties of PMO-Polysorbate complexes warrant detailed ADME (absorption, distribution, metabolism, and excretion) profiling with diverse Polysorbates to optimize therapeutic outcomes. Undoubtedly, the present research provides a vital baseline for studying PPMOs (peptide-conjugated PMOs) and other advanced oligonucleotides, such as GMO-PMO (Guanidinium-Morpholino oligonucleotide-PMO), TMO-PMO (Thiomorpholino oligonucleotide-PMO), and DNA-PMO chimeras, which bear the same morpholino linkages along with some charged ones.^7^^,^^8^^,^^9^

**Broader Outlook:** This study transforms the “invisible strength” of Polysorbates into a measurable, exploitable tool. It sets the stage for next-generation precision medicine and cellular therapies by integrating surfactant science into oligonucleotide drug design.

## Future directions

Future studies should aim to explore “surfactant cocktails” (mixtures of Polysorbate 20 and 80) to leverage differences in binding energies and molecular sizes for enhanced stabilization. Furthermore, as the field moves toward more complex delivery vehicles, such as lipid nanoparticles (LNPs) or ligand-conjugated oligonucleotides, the ability to decouple charge interactions from purely hydrophobic and hydrophilic forces will be essential. Tailored Polysorbate blends could enable high-concentration fills, subcutaneous delivery, or combination therapies—potentially slowing DMD progression and extending applications to oncology and viral diseases. This study transforms the “invisible strength” of Polysorbates into a measurable, exploitable tool. It sets the stage for next-generation precision medicine and cellular therapies by integrating surfactant science into oligonucleotide drug design.

## Critical perspective

While the study is comprehensive, a few points merit further exploration:

**Stoichiometry beyond 1:1 complexes**: The simulations focus on single surfactant-PMO complexes, but in real formulations, multiple surfactant molecules may interact simultaneously. Extending simulations to higher stoichiometries could reveal cooperative or competitive binding effects.

**Sequence dependence**: The study emphasizes preferential interaction of Polysorbates with purine base faces, providing a foundation for exploring interactions with nucleobase-functionalized morpholinos.^10^ Nevertheless, broader sequence diversity (e.g., GC-rich vs. AT-rich regions) might influence surfactant binding patterns.

**Chimeric backbone dependence:** The study mainly focuses on PMOs containing fully phosphorodiamidate backbones. However, recent advances in morpholino gapmers (PMO-phosphorothioate DNA-PMO) indicate their therapeutic potential in the near future. This opens up an avenue for exploring interactions between surfactants and morpholino gapmers to enhance their biological efficacy.

**Biological context**: The work is primarily physicochemical. Future studies could assess whether surfactant-stabilized PMOs exhibit altered cellular uptake, biodistribution, antisense activity, and toxicity profiles.

## Acknowledgments

S.S. thanks 10.13039/501100001843SERB SUPRA (SPR/2023/000358) for financial support.

## Declaration of interests

The authors wrote this commentary in a personal capacity, reflecting their own professional expertise and viewpoints. The authors declare no competing interests.
